# *miR-372* inhibits p62 in head and neck squamous cell carcinoma *in vitro* and *in vivo*

**DOI:** 10.18632/oncotarget.3340

**Published:** 2015-01-21

**Authors:** Li-Yin Yeh, Chung-Ji Liu, Yong-Kie Wong, Christine Chang, Shu-Chun Lin, Kuo-Wei Chang

**Affiliations:** ^1^ Institute of Oral Biology, National Yang-Ming University, Taipei, Taiwan; ^2^ Department of Dentistry, National Yang-Ming University, Taipei, Taiwan; ^3^ Department of Dentistry, Taipei Mackay Memorial Hospital, Taipei, Taiwan; ^4^ Department of Dentistry, Taichung Veterans General Hospital, Taichung, Taiwan; ^5^ Department of Stomatology, Taipei Veterans General Hospital, Taipei, Taiwan

**Keywords:** Carcinoma, Hypoxia, Invasion, miR-372, Mouth

## Abstract

Here we showed that exogenous *miR-372* expression and knockdown of p62 (sequestosome1 or SQSTM1), both increased migration of head and neck squamous cell carcinoma (HNSCC) cells. p62 induced phase II detoxification enzyme NADPH quinone oxidoreductase 1 (NQO1), which decreased ROS levels and cell migration. Also, *miR-372* decreased p62 during hypoxia, thus increasing cell migration. Levels of *miR-372* and p62 inversely correlated in human HNSCC tissues. Plasma levels of *miR-372* was associated with advanced tumor stage and patient mortality. Both plasma and salivary *miR-372* levels were decreased after tumor resection. We conclude that *miR-372* decreases p62, thus increasing ROS and motility in HNSCC cells.

## INTRODUCTION

Head and neck squamous cell carcinoma (HNSCC), is one of the prevalent neoplasms worldwide [1-3]. Many risk factors, including smoking, alcohol consumption, areca chewing and HPV infection, can affect the genesis of HNSCC [4, 5]. Although there are several treatment modalities being developed for HNSCC treatment, a low 5-year survival rate of HNSCC patients has not improved over the past decades [2, 6]. To develop new strategies that will intercept HNSCC development, a molecular understanding of HNSCC pathogenesis is important. Over the last decade, many studies have indicated that non-protein-coding miRNAs are highly associated with human tumor promotion and progression [7]. miRNAs are able to function as oncogenes or as tumor suppressors, and it should be possible to use them as diagnostic biomarkers or therapeutic targets of malignancies including HNSCC [1-3, 6-13].

Hypoxia is a feature of most tumors during their late stages. Imbalance between oxygen consumption and oxygen delivery in tumor tissue may give rise to a hypoxic microenvironment, which in turn stimulates gene responses that are associated with chemo-resistance or radio-resistance, angiogenesis, invasion and metastasis, all of which benefit tumor progression [14-16]. In a hypoxia status, certain transcription factors, namely the hypoxia-inducible factors (HIFs), are the master gene controllers that activate the transcription program promoting the survival of cells in this adverse microenvironment [17, 18]. It has been reported that hypoxia is involved in regulating *miR-210*, *miR-34a*, *miR-17/20a* and others miRNAs that contribute to tumor growth, the epithelial-mesenchymal transition (EMT) and myeloid leukemic cell differentiation via HIF1α [19-22]. Hypoxia also up-regulated Bcl2/adenovirus E1B 19 kDa interacting protein 3 (BNIP3) that triggers mitochondrial autophagy [16]. In addition, we also identified that *miR-31* is able to activate HIF1α as part of HNSCC pathogenesis by targeting its inhibitor [1].

*miR-372*, *miR-373, miR-302, miR-520* and some other miRNAs are members of *miR-93* family. The *miR-372* and *miR-373* miRNA cluster were originally found to be associated with stemness in embryonic cells. It was then found that they act as oncogenes during the tumorigenesis of human testicular germ cell tumors by concomitant targeting of LATS2 and CD44 in order to overcome senescence and to promote metastasis, respectively [23]. They are up-regulated in hepatocellular carcinoma, colorectal carcinoma (CRC), glioma, testicular germ cell tumors and gastric carcinoma [23-28]. Expression of *miR-372* has been correlated with a poor prognosis and aggressive tumor growth [27]. Furthermore, up-regulation of *miR-372/miR-373* has been found in HNSCC tissues during previous screenings [1, 29]. A recent study identified that *miR-372* affects esophageal and gastric carcinogenesis via an inhibition of LATS2 expression [25, 28]. Furthermore, β-catenin transactivates *miR-372/miR-373*, which then target DKK1 [30]. Moreover, a study indicated that *miR-372* is a hypoxia up-regulated miRNA and that it targets the tumor suppressor RECK during pathogenesis [22]. In contrast, *miR-372* has been shown to be down-regulated in cervical carcinoma and is able to target CDK2 [31].

p62 (also called sequestosome1 or SQSTM1) is an ubiquitin-binding protein that chaperones protein aggregates to the lysosome for degradation during autophagy, and is up-regulated by autophagy inhibition [4, 32, 33]. It is also a multidomain protein that interacts with other molecules and as a result has a profound impact on signal regulation [34]. p62 binds to the Kelch-like ECH-associated protein 1 (Keap1) in competition with Nrf2, which results in the stabilization and activation of Nrf2; this induces the transcription of antioxidant genes such as phase II enzyme NAD(P)H quinone oxidoreductase 1 (NQO1) and haem oxygenase-1 in order to maintain reactive oxygen species (ROS) homeostasis [35]. However, p62 is also able to modulate ROS through mTOR pathway, which bypasses the requirement of NQO1, in stromal fibroblast [36]. Multiple molecular mechanisms are known to take part in regulating cancer cell migration [1-3, 12, 15, 37-41]. In this study, we provide novel clues as to how *miR-372* targets p62, which, in turn, enhances the mobility of HNSCC cells.

## RESULTS

### *miR-372* promotes the migration of HNSCC cells and targets p62

**Fig.1:**
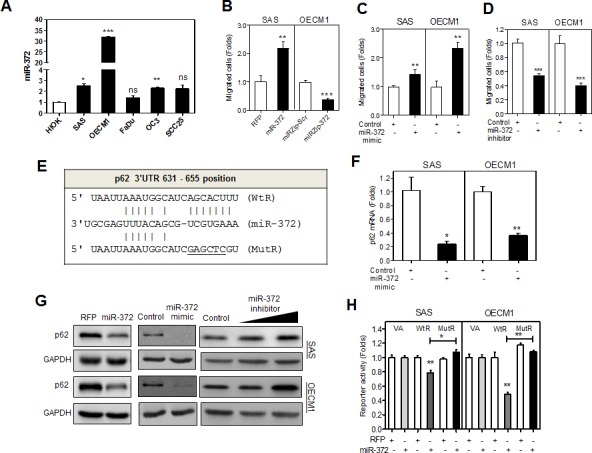
*miR-372* enhances migration of HNSCC cells and targets p62 (A) qRT-PCR analysis of *miR-372* expression in HNSCC cell lines and HIOK cell. All cell lines had *miR-372* expression equal to or higher than HIOK. (B - D) Association between the *miR-372* expression and migration in HNSCC cells. (B) SAS-miR-372 cell subclone exhibited the enhancement of migration relative to control (SAS-RFP). OECM1-miRZip-372 cell subclone exhibited the decrease of migration relative to control (OECM1-miRZip-Scr). (C, D) Treatment with *miR-372* mimic (in C) and *miR-372* inhibitor (in D) significantly increased and decreased the migration of HNSCC cells, respectively. (E) Schematic diagram to show the complimentarity between *miR-372* and the 3′UTR of the *p62* gene. (F) qRT-PCR analysis for *p62* mRNA expression. Exogenous *miR-372* expression decreased *p62* mRNA expression. (G) Western blot analysis. Exogenous *miR-372* expression decreased p62 protein expression (Lt and Middle), while *miR-372* inhibition increased p62 expression (Rt) in HNSCC cells. (H) Reporter assay. WtR and MutR reporters designated in (E), together with a vector alone (VA) reporter were used. The underline designates mutated nucleotides, which includes *Sac*I restriction enzyme digestion site. The mutations in the targeted sequences abolished the complimentarity with seed sequences. The analysis indicated that there was significant repression of WtR activity in HNSCC cells, while this reduction in activity was reversed with MutR. Data shown were mean ± SE. *ns*, not significant,*, *p* <0.05; **, *p* <0.01; ***, *p* <0.001; Mann-Whitney test.

Our previous study demonstrated that *miR-372* was up-regulated in HNSCC tissue samples [1]. To further investigate the functional roles of *miR-372* in head and neck pathogenesis, the endogenous *miR-372* expression in various head and neck keratinocytes was analyzed. Human hTERT immortalized oral keratinocyte (HIOK) and HNSCC cells exhibited different levels of endogenous *miR-372* expression. OECM1 cell line had the highest level of *miR-372* expression, while SAS cell line exhibited *miR-372* expression similar to other HNSCC cell lines (Fig. 1A). We established SAS-miR-372 and OECM1-miR-372 cell subclones expressing exogenous *miR-372* and SAS-miRZip-372 and OECM1-miRZip-372 cell subclones harboring stable suppression of *miR-372* by lentiviral infection, sorting or selection of cells. The stable *miR-372* expression enhanced the migration of SAS cells and the stable *miR-372* inhibition reduced the migration of OECM1 cells (Fig. 1B). However, the exogenous *miR-372* expression or *miR-372* inhibition did not cause changes in cell proliferation (Fig. S1A). To exclude any confounding effect driven by the passenger strand of the *miR-372* duplex, SAS and OECM1 cells were treated with *miR-372* mimic, the passenger strand of which had been silenced by modification. The treatment resulted in the expression of *miR-372*, but not of *miR-373* or miR-372* (detailed analysis not shown). This did increase the migration of cells (Fig. 1C), but had no influence on cell growth or the responses to cisplatin treatment (Fig. S1B). The inhibition of *miR-372* with the treatment of mirVana^TM^ *miR-372* inhibitor decreased the migration of cells (Fig. 1D), but it did not affect cell proliferation (Fig. S1C).

TargetScan and PicTar *in silico* modules predicted that p62 might be an unreported target of *miR-372* (Fig. 1E). qRT-PCR analysis indicated that *p62* mRNA expression was significantly down-regulated following the treatment with *miR-372* mimic in both SAS and OECM1 cells (Fig. 1F). SAS-miR-372, OECM1-miR-372 cell subclones, and both SAS and OECM1 cells treated with *miR-372* mimic were subjected to Western blot analysis to show the down-regulation of p62 following increased expression of *miR-372* (Fig. 1G, Lt and Middle). On the contrary, cells treated with *miR-372* inhibitor exhibited up-regulation of p62 (Fig. 1G, Rt). To determine whether *miR-372* is able to suppress p62 expression through direct binding to its 3′UTR, we transfected cells with the wild type reporter (WtR) and mutant reporter (MutR) plasmids (see schema in Fig. 1E). Luciferase activity assays indicated that *miR-372* was able to repress the activity of WtR by binding to the wild type target sequences in the 3′UTR, while mutation of these wild type sequences removed this repression in both SAS-miR-372 and OECM1-miR-372 cell subclones (Fig. 1H).

### *miR-372* enhances cell migration by targeting p62

**Fig.2:**
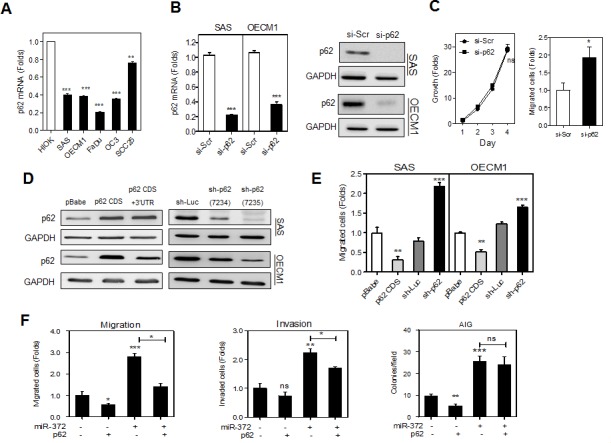
p62 inhibits the migration of HNSCC cells (A) qRT-PCR analysis of *p62* mRNA expression in HNSCC cell lines and a HIOK cell. All cell lines tested had *p62* mRNA expression lower than HIOK. (B) Transient knockdown of p62. Lt, *p62* mRNA expression Rt, p62 protein expression. The treatment with si-p62 oligonucelotide remarkably down-regulated endogenous p62 expression in HNSCC cells. (C) Phenotypic analysis of OECM1 cells. Lt, growth assay; Rt migration assay. Down-regulation of p62 was associated with an increase in the cell migration of OECM1 cells. (D) Generation of stable HNSCC cell subclones with exogenous p62 expression (Lt) and knockdown of p62 expression (Rt). The p62 CDS cell subclone had the highest p62 expression, while the sh-p62 (7235) cell subclone had the lowest p62 expression. (E) Migration analysis. Cell migration was decreased in the p62 CDS cell subclone, but increased in the sh-p62 cell subclone of HNSCC cells. (F) Rescue of the *miR-372* induced phenotypes by p62 expression in OECM1 cells. The p62 CDS+3′UTR cell subclone was treated with *miR-372* mimic and then migration (Lt), invasion (Rt) and AIG (Lower) were analyzed. The results indicated that p62 reverted the increase in migration and invasion, but not AIG, mediated by *miR-372*. Data shown are mean ± SE. *ns*, not significant,*, *p* <0.05; **, *p* <0.01, ***, *p* <0.001; Mann-Whitney test or two-way ANOVA test.

The expression of *p62* mRNA in HNSCC cells was lower than the HIOK analyzed (Fig. 2A). Next we investigated the phenotypes of HNSCC cells following knockdown of p62. HNSCC cells treated with si-p62 and control oligonucleotide were analyzed. The down-regulation of *p62* mRNA expression (Fig. 2B, Lt) and protein expression (Fig. 2B, Rt) was noted. si-p62 treatment did not affect cell proliferation (Fig. 2C, Lt), but it promoted cell migration of OECM1 cells (Fig. 2C, Rt). We further established SAS and OECM1 cell subclones with exogenous p62 expression or knockdown of p62 expression. Western blot analysis revealed that there was increased p62 expression in p62 coding sequence (CDS) cell subclones, but this increase became weaker in p62 CDS+3′UTR stable cell subclones, which contains the *miR-372* target site in the 3′UTR (Fig. 2D, Lt). As OECM1 cells expressed *miR-372* at a much higher level than SAS cells, the decrease in exogenous p62 expression via targeting of *miR-372* was particularly obvious in these cells. Since sh-p62 (7235) cell subclones exhibited more conspicuous knockdown of p62 than sh-p62 (7234) cell subclones (Fig. 2D, Rt), sh-p62 (7235) cell subclones were subjected to subsequent analysis. The migration capability of HNSCC cells seemed to be related to p62 expression level in a reverse manner (Fig. 2E). To further confirm that *miR-372* enhances cell mobility by targeting p62, p62 CDS+3′UTR cell subclones of OECM1 were treated with *miR-372* mimic. The results indicated that the increased migration (Fig. 2F, Lt) and invasion (Fig. 2F, Middle), other than anchorage-independent growth (AIG) (Fig. 2F, Rt), which was induced by *miR-372* expression, was significantly attenuated by p62 expression. Although *miR-372* also targets LATS2 in HNSCC cells, the knockdown of LATS2 did not affect HNSCC cell migration (detailed analysis not shown).

### p62 induced NQO1 expression is able to attenuate migration

**Fig.3:**
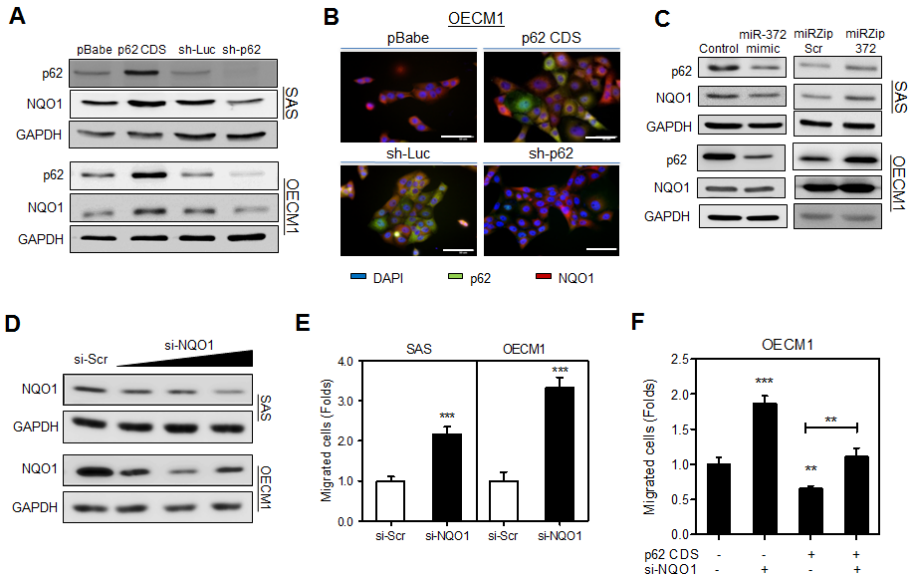
p62 regulates cell migration through NQO1 modulation (A, C, D) Western blot analysis. (A) It showed that NQO1 protein expression level was concordant with p62 protein level in various HNSCC cell subclones. (B) IF. It showed the co-localization of increased NQO1 fluorescence with exogenous p62 (Upper), and the decrease of co-localized p62/NQO1 fluorescence following the knockdown of p62 (Lower), mainly in cytosol of OECM1 cells. The exposure time of the images in lower panel was much longer than that in the upper panel. Bars, 20 μm. (C) Exogenous *miR-372* expression decreased NQO1 expression (Lt), and *miR-372* inhibition increased NQO1 expression (Rt) in SAS cells. The changes of NQO1 in OECM1 cells was not so eminent. (D) Knockdown of NQO1. It showed the down-regulation of NQO1 in cells following treatment with the si-NQO1 oligonucleotide with increasing dosage. (E, F) Migration analysis. (E) The migration of HNSCC cells increased following the knockdown of NQO1. (F) Knockdown of NQO1 in HNSCC cells rescued the p62 induced effects on migration. The decrease of migration caused by p62 was reverted on treatment with si-NQO1. Data shown were mean ± SE. **, *p* <0.01, ***, *p* <0.001; Mann-Whitney test.

The protein level of NQO1 in HNSCC cells is highly related to p62 protein level following the exogenous expression or knockdown of expression as shown by Western blot analysis (Fig. 3A). IF analysis also revealed a concordance of p62 and NQO1 immunoreactivity in the cytosol of OECM1 cell subclones (Fig. 3B). In HNSCC cells, the exogenous *miR-372* expression is associated with the down-regulation of both p62 and NQO1 (Fig. 3C, Lt), and the *miR-372* inhibition is associated the up-regulation of p62 (Fig. 3C, Rt). The up-regulation of NQO1 associated with *miR-372* inhibition was eminent in SAS cells, while it was not eminent in OECM1 cells. Using si-NQO1 oligonucleotide to knock down NQO1 (Fig. 3D), we further identified that there was an increase in cell migration in the HNSCC cells (Fig. 3E). Lastly, we showed that a reduction in OECM1 cell migration could be achieved by means of p62 expression, which can be rescued by the knockdown of NQO1 (Fig. 3F). The findings indicate that p62 induced NQO1 expression is able to attenuate migration.

### Involvement of *miR-372* and p62 in the genesis of ROS

**Fig.4:**
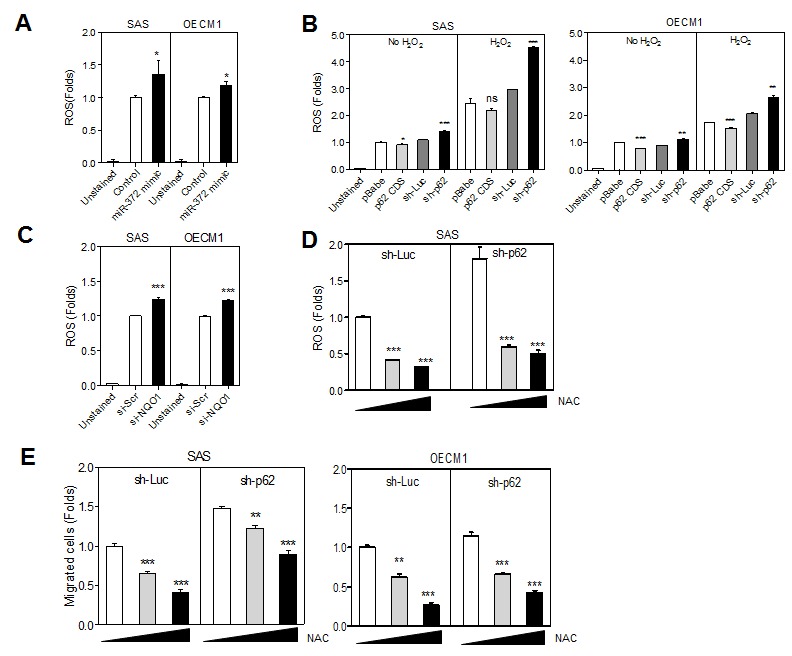
*miR-372*-p62 modulates ROS and migration in HNSCC cells (A-D) Detection of ROS. (E) Migration assay. (A) The treatment with *miR-372* mimic slightly increased the ROS in cells. (B) The endogenous ROS and the H_2_O_2_ stimulated ROS are related to the p62 protein level in various HNSCC cell subclones. Lower p62 expression was associated with higher ROS in cells. (C) ROS was also increased following the knockdown of NQO1. (D) In sh-Luc and sh-p62 cell subclones of SAS cells, the treatment with NAC reduced ROS in a dose-dependent manner (E). NAC treatment also reduced the migration of HNSCC cells in a dose-dependent manner. Data shown were mean ± SE. *ns*, not significant; *, *p* <0.05; **, *p* <0.01, ***, *p* <0.001; Mann-Whitney test.

Exogenous *miR-372* expression was associated with increased ROS in HNSCC cells (Fig. 4A). In addition, the level of p62 expression was also generally related to ROS genesis either in the absence of stimulation or in the presence of H_2_O_2_ stimulation (Fig. 4B). The down-regulation of p62 was found to increase endogenous and H_2_O_2_ stimulated ROS. Knockdown of NQO1 increased ROS in HNSCC cells (Fig. 4C). With the treatment of N-acetyl-L-cysteine (NAC), the endogenous ROS and the ROS evoked by knockdown of p62 was attenuated (Fig. 4D). It was also noted that the ROS level was correlated with the competence of migration of HNSCC cells as NAC treatment attenuated the migration of control cell subclones and cell subclones with p62 knockdown (Fig. 4E).

### Hypoxia induced migration can be mediated through the *miR-372*-p62 cascade

**Fig.5:**
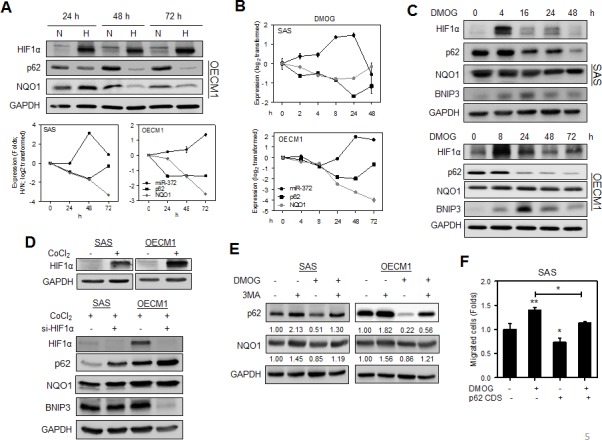
Hypoxia induced migration is attenuated by p62 (A) HNSCC cells were grown in hypoxia culture for 24 h - 72 h. Upper, Western blot analysis revealed that p62 and NQO1 protein expression decreased gradually during hypoxia culture in OECM1 cells. Lower, qRT-PCR analysis revealed highly significant *miR-372* up-regulation together with down-regulation of *p62* and *NQO1* mRNA expression at 48 h for SAS cells, and at 72 h for OECM1 cells. (B, C) HNSCC cells treated with 500 μM DMOG. (B) qRT-PCR analysis. (C) Western blot analysis. DMOG treatment induced *miR-372* up-regulation and down-regulation of the mRNA expression of *p62* and *NQO1*. This regulation was particularly eminent for SAS cells at 24 h and OECM1 at 48 h (in B). The treatment drastically up-regulated HIF1α protein expression, and it resulted in the progressive down-regulation of p62 protein expression. The down-regulation of p62 and NQO1 for SAS cells occurred 16 h to 48 h after treatment. BNIP3 expression was induced 4 h or 8 h after treatment, which reached peak after HIF1α peaked. (D) HNSCC cells treated with 200 μM CoCl_2_. Upper, Western blot analysis showed the expression of HIF1α following the treatment with CoCl_2_ for 24 h in HNSCC cells. Lower, Knockdown of HIF1α using si-HIF1α reversed the p62 down-regulation and BNIP3 up-regulation that was modulated by CoCl_2_ for 48 h. The change in NQO1 expression was not eminent. (E) Blockage of autophagy. Cells were treated with 500 μM DMOG and/or 100 mM 3MA for 16 h. The results indicated that the presence of DMOG induced p62 down-regulation under the circumstances where there was autophagy blockage. The down-regulation of NQO1 protein expression was also present despite that it was not so eminent. Numbers were normalized values. (F) Expression of p62 reverted the DMOG induced migration in SAS cells. Data shown were mean ± SE. *, *p* <0.05, **, *p* <0.01; Mann-Whitney test.

HNSCC cells were cultured under hypoxia conditions for 24 h - 72 h. Western blot analysis revealed that both p62 protein expression and NQO1 protein expression decreased gradually over the culture period (Fig. 5A, Upper). *miR-372* up-regulation occurred earlier on 48 h in SAS cells than in OECM1 cells. The mRNA expression of *p62* and *NQO1* was down-regulated during hypoxia culture and it was generally opposite to the status of *miR-372* expression (Fig. 5A, Lower). The changes in mRNA expression of *p62* and *NQO1* appeared similar to their protein expression status under hypoxia culture. Cells were also treated with dimethyloxaloylglycine (DMOG) or cobalt chloride (CoCl_2_) to induce HIF1α expression. Following the DMOG treatment, *miR-372* expression was gradually up-regulated to a maximal level at 24 h for SAS cells and at 48 h for OECM1 cells. The mRNA expression of *p62* and *NQO1* was generally down-regulated during DMOG treatment and this was opposite to the effect on *miR-372* expression (Fig. 5B). Following DMOG treatment, the gradual down-regulation of p62 protein and the induction of the autophagy marker BNIP3 after HIF1α expression was seen (Fig. 5C). As the knockdown of HIF1α expression after treatment with si-HIF1α reversed the p62 down-regulation and autophagy induction during CoCl_2_ treatment, the changes in p62 and BNIP3 were considered secondary to the changes in HIF1α expression (Fig. 5D). The genesis of autophagy might also result in p62 down-regulation and therefore we treated with 3MA to block autophagy [4]. After the blockage of autophagy by 3-methyladenine (3MA), the hypoxia-induced p62 down-regulation still existed, which substantiates the involvement of hypoxia induced *miR-372* expression in the attenuation of p62 expression, which is independent from autophagy (Fig. 5E). Furthermore, hypoxia induced cell migration was attenuated in SAS cells when there was exogenous expression of p62 (Fig. 5F). These findings indicate that hypoxia induced migration of HNSCC cells are able to be mediated by the *miR-372*-p62 regulatory cascade.

### Contrasting expression of p62 compared to *miR-372* in HNSCC tissue samples

**Fig.6:**
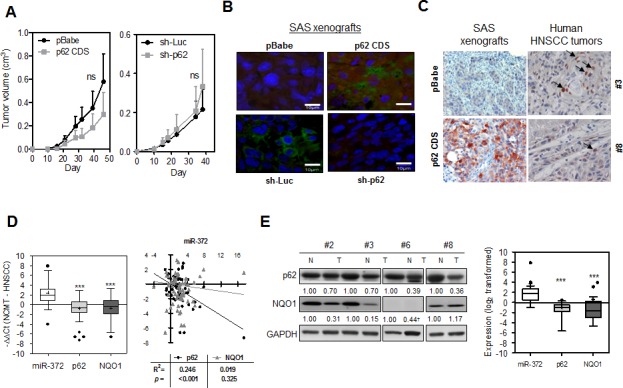
Expression of *miR-372*, p62 and NQO1 in HNSCC tissues (A) Growth curves of subcutaneous SAS cell xenografts. Lt, p62 expression cell subclones; Rt, Knockdown of p62 cell subclones. At least five mice in each group. (B) IF analysis of p62 using SAS xenografts. Both the endogenous and exogenous p62 were localized in cytosol. The exposure time of the images in lower panels was much longer than that in the upper panels. Bars, 10 μm. (C) IHC analysis for p62. Lt, SAS xenografts (x100). Weak endogenous and strong exogenous p62 immunoreactivity mainly localized in the cytosol was seen in tumor cells. Rt, two distinctive human HNSCC tumors (x200). Both cytosolic and nuclear staining can be seen in these tumors. In some cells (arrow indicated), the nuclear p62 immunoreactivity was very intensive. (D) Lt, qRT-PCR analysis of HNSCC tissue pairs. Box and Whiskers plot. Y-axis, −ΔΔCt; +, mean value; horizontal line, medium value. Up-regulation of *miR-372* and down-regulation of *p62* and *NQO1* mRNA expression can be seen. Rt, Correlation analysis between *miR-372* expression (X-axis) and the mRNA expression of *p62* or *NQO1* (Y-axis) in HNSCC tissue pairs. *miR-372* expression levels are inversely correlated with *p62* mRNA expression. (E) Lt, Western blot analysis of representative tissue pairs. Numbers were normalized values. ^†^, questionable values. The values were not subjected to quantification. Rt, Box and Whiskers plot illustrating *miR-372* expression and the protein expression of p62 and NQO1 in tissue pairs. Y-axis, log_2_ transformed expression level. +, mean value; horizontal line, medium value. Three sample pairs exhibited questionable NQO1 quantification (for example, case #6 labeled † in Lt panel) were excluded for analysis. *ns,* not significant, *, *p* < 0.05; **, *p* <0.01; ***, *p* <0.001; two-way ANOVA test or Mann-Whitney test.

SAS cell subclones were injected subcutaneously into the flank of nude mice to evaluate the association between p62 expression and the xenografic tumor induction. The analysis of tumor volume indicated that the tumorigenesis of the SAS cells was not significantly affected by changes in p62 expression (Fig. 6A). IF were performed on tissue sections of the SAS xenografts and revealed an increase in exogenous p62 immunoreactivity in the cytosol of p62 CDS tumor cells compared to the control cells. In addition, the endogenous p62 was also found mainly localized in the cytosol of the tumor cells, and it can be knocked down by sh-p62 (Fig. 6B). Nevertheless, preliminary IHC analyses revealed the presence of both cytosolic and nuclear staining in human HNSCC tumors (Fig. 6C, Rt) in contrast to the cytosolic p62 staining in SAS xenografts (Fig. 6C, Lt). Furthermore, the vast majority of tumors examined seemed to have low intensity of such staining (Fig. 6C). This raised concerns as to the specificity of the IHC staining. Thus, HNSCC tumors and their paired non-cancerous matched tissue samples (NCMTs) were analyzed to explore the simultaneous expression of *miR-372*, *p62* and *NQO1* (Table S2). qRT-PCR analysis of 66 sample pairs indicated an average change of –ΔΔCt of 2.37, −0.83 and −0.76 for *miR-372*, *p62* and *NQO1*, respectively, in HNSCC samples relative to NCMT samples (Fig. 6D, Lt). Significant opposite effects on the expression of *miR-372* and *p62*, and of *miR-372* and *NQO1* could be seen. There was an inversion correlation between the expression of *miR-372* and *p62* mRNA in HNSCC tissues analyzed (Fig. 5D, Rt). We also performed Western blot analysis using 25 pairs of tissues available for analysis (Fig. 6E, Lt). The analysis showed that there was down-regulation of the p62 and NQO1 signals in the tumor tissues relative to paired NCMT samples. Faint or scanty NQO1 signals were identified in some sample pairs. Quantification indicated a significant down-regulation of the protein levels of p62 and NQO1 compared to *miR-372* expression across this tissue subset (Fig. 6E, Rt).

### High levels of plasma *miR-372* is associated with the progression of HNSCC and higher mortality of patients

**Fig.7:**
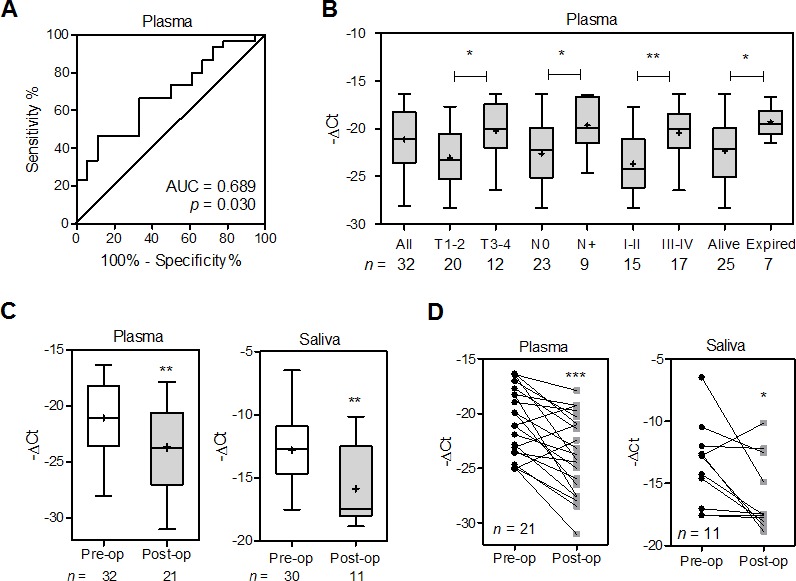
Increased plasma *miR-372* defines HNSCC progression and worse prognosis (A) ROC analysis of plasma *miR-372*. Comparison was carried out across plasma samples from control subjects and those from HNSCC patients. (B, C). Box and Whiskers plots illustrating *miR-372* in the plasma or saliva samples of HNSCC patients. X-axis, clinical settings or operative status; Y-axis, −ΔCt. Patients having a higher plasma *miR-372* were found to have larger primary tumors, nodal metastasis, a more advanced stage and higher mortality during follow-up. Plasma and salivary *miR-372* decreased after tumor resection. Mann-Whitney test. (D) Before and after plot of paired plasma samples (Lt) and paired saliva samples (Rt) collected pre-operatively (Pre-op) and post-operatively (Post-op) from patients. It showed the decline of *miR-372* in patient's plasma and saliva after tumor resection. Wilcoxon signed rank test. *, *p* < 0.05; **, *p* <0.01; ***, *p* <0.001.

To examine the feasibility of using the plasma level of *miR-372* as a diagnostic marker, plasma samples were collected from HNSCC patients and controls. qRT-PCR analysis indicated a mean –ΔCt of −21.1 in patients with HNSCC in relation to −22.9 in controls. ROC analyses indicated that the plasma *miR-372* level had a predictive power of 0.69 for distinguishing malignant from non-malignant states (Fig. 7A). Patients with larger primary tumors, nodal metastasis and an advanced clinical stage exhibited higher *miR-372* level in their pre-operative plasma than the remaining group of patients. This increase in *miR-372* was also associated with a higher mortality among the HNSCC patients (Fig. 7B). There was a decline in *miR-372* when HNSCC patients' post-operative plasma was compared to their pre-operative plasma, which suggests that the origin of the plasma *miR-372* is the tumor tissue within the patients (Fig. 7C, Lt; 7D, Lt). Thus, plasma *miR-372* would seem to be validated as a marker for HNSCC. qRT-PCR analysis also detected the salivary *miR-372* level with a mean –ΔCt of −12.8 in HNSCC patients. A decline of *miR-372* was seen in patients' post-operative saliva compared to pre-operative saliva (Fig. 7C, Rt). Paired analysis also indicated that 8 out of 11 (72.7%) patients exhibited the conspicuous decrease of salivary *miR-372* after tumor resection (Fig 7D, Rt). It is likely that the salivary *miR-372* in patients derived from tumor tissues. Overall, the findings of this study indicate that *miR-372* is able to evoke migration and increase ROS via targeting of p62, which may decreases NQO1 expression then, and the circulatory *miR-372* could be HNSCC tumor marker.

Additional results are presented in the supplementary material.

## DISCUSSION

Oxygen deficiency leading to hypoxia is a common feature of solid tumors during progression and this includes HNSCC [16]. HIF1α induced by hypoxia turns on a transcription program that promotes an aggressive tumor phenotype and resistance to therapies via triggering of critical genes [16]. The *miR-372* and *miR-373* miRNA cluster were originally associated with stemness in embryonic cells and oncogenicity in human testicular germ cell tumors via a concomitant targeting of LATS2 and CD44 [23]. Recently, *miR-372/miR-373* have been found to be up-regulated in response to hypoxia via HIF1α and TWIST1 in SW620 CRC cells [22]. In addition, β-catenin is also known to up-regulate the expression of the *miR-372/miR-373* cluster through promoter transactivation [30]. This study further identified that *miR-372* was up-regulated by hypoxia and by HIF1α up-regulation in head and neck keratinocytes. Since areca, an oral carcinogen, up-regulates HIF1α in HNSCC cells [4] and nicotine is also known to markedly up-regulated HIF1α expression in nasopharyngeal carcinoma cells [42], it seems likely that *miR-372* contributes to human HNSCC genesis via stimulation by habitual use of carcinogenic substances.

Although p62 is a substrate adaptor that plays critical roles in autophagy by transferring elements into autophagosome, studies also have indicated modulation of p62 within the NFκB, mTOR and Wnt signaling pathways, indicated that p62 acts as multifunctional protein [35, 36]. It was also clear that p62, together with other molecules that interact with it, can be degraded in the autophagosome during autophagy [4, 32]. ROS may up-regulate p62 expression through promoter activation [43]; however, no report has ever addressed the regulatory effect of a miRNA on p62. This study presents novel clues showing that *miR-372* targets p62 by virtue of the binding its 3′UTR site and that this down-regulates p62, which then increases the mobility of HNSCC cells. Although it has been reported that p62 mediates apoptosis and cisplatin resistance by preventing apoptosis in various cell types that harbor a range of different molecular networks [39, 44], p62 was shown in this study to be functionally irrelevant to cell viability and to have no effect on the response to cisplatin treatment of HNSCC cells. Instead, we identified a decrease of HNSCC cells that was mediated by p62. Furthermore, expression of p62 is able to attenuate the migration induced by *miR-372*. Although p62 has been found to modulate the increased migration of glioblastoma stem cells [45], multiple functions of p62 seem to be present depending on the cellular circumstances [36, 46]. As p62 is also able to interact with cytoskeletal components, it would be interesting to explore whether p62 inhibits migration by disrupting these molecules [38].

p62 has recently been reported binds to Keap1, which then results in the release of Nrf2 [43]. Nrf2 activates downstream antioxidant proteins and NQO1 to protect against oxidant stress and to protect cells from DNA damage. Thus, lines of evidence seem to support the hypothesis that NQO1 plays a role in cancer prevention [34]. However, a recent study has suggested that knockdown of p62 does not affect the Nrf2-Keap1 pathway in HNSCC cells [47]. Using both expression and knockdown approaches in distinctive HNSCC cells, we identified concordance between p62 and NQO1 expression. In addition, changes of expression in p62 and NQO1 were rather synchronized during hypoxia induction. These findings substantiate the presence of a p62-Nrf2-NQO1 regulatory cascade in HNSCC cells. However, the influence of *miR-372* on NQO1 expression was somewhat redundant in OECM1 cell line as more complicated factors could be involved. We identified the cytosolic localization of p62 in HNSCC cell lines and their xenografts. The findings are in agreement with the human prostate tumors and rodent pancreatic carcinomas carrying autophagy impairment, which exhibit intense cytosolic p62 immunoreactivity [33, 36]. The pathological significance of nuclear p62 being identified in HNSCC tumor requires further elucidation [47]. Since the reduction in migration that is mediated by p62 can be rescued by the knockdown of NQO1, this further supports a role for the *miR-372*-p62-NQO1 cascade in the progression of HNSCC. Various lines of evidence support the hypothesis that ROS may participate in the regulation of Rho family members and modulate cytoskeletal organization or that ROS may activate other signals to increase migration [41, 48]. NQO1 was reported to attenuate ROS and the proliferation of vascular endothelial cells [49]. Of note, the regulation of p62 on mTOR can also modulate ROS genesis, which bypasses the requirement of NQO1 [36]. Although we showed the increase of ROS and mobility of HNSCC cells after knocking known NQO1, whether NQO1 plays a direct role in repressing the mobility of HNSCC cells is an important issue that needs to be elucidated.

Hypoxia induces the migration of HNSCC cells [1]. This study has demonstrated the occurrence of a consequential change in the *miR-372*-p62-NQO1 axis following the induction of hypoxia. This molecular mechanism underlies the increased migration of HNSCC in a hypoxia microenvironment. Although *miR-372* is known to act as either an oncogenic miRNA or as a suppressor miRNA in various human malignancies [23, 26, 31], *miR-372* is known to be up-regulated in HNSCC in earlier studies [1, 29]. The fact that a large fraction of HNSCC tumor cells exhibiting high HIF1α expression could be one of the reasons for this [1, 17]. We further validated the concomitant down-regulation of p62 and NQO1 that accompanies *miR-372* up-regulation in HNSCC tissues and that *miR-372* is able to modulate the migration of HNSCC cells by suppression in this study. It is well known that hypoxia induced the EMT, which facilitated the invasiveness of HNSCC [15] and this study provides another clue demonstrating that *miR-372* expression, when elicited by hypoxia, also mediates cell mobility by targeting p62 and its downstream detoxification cascade.

Plasma miRNAs are able to provide noninvasive markers for HNSCC [3, 9, 29]. Our clinical analysis indicated that the plasma level of *miR-372*, which is most likely derived from tumors, is also associated with the progression of HNSCC. *miR-372*, as a member in a panel of miRNAs, has been suggested to be suitable for predicting lung carcinoma from sputum samples [11]. In addition, other miRNA profile in sputum can assist the early diagnosis of squamous lung carcinoma [10]. Our preliminary analysis also detected the tumor-derived *miR-372* in the saliva samples of HNSCC patients. To develop plasma markers for HNSCC using a panel of miRNAs, including *miR-372*, could be a promising strategy [6]. *miR-372* expression is known to repress several target genes that alter cellular phenotypes [23, 30, 31]; this suggests that the uncovering of additional *miR-372* targets and an exploration of the pluripotent actions of *miR-372* across different subset of tumors is likely to have important implications. As p62 is involved in pluripotential modulation of autophagy, tumorigenesis and the modification of tumor microenvironment [32, 33, 36], this study was only able to address its roles in counteracting oxidative stress during pathogenesis. A further understanding of the molecular implications associated with *miR-372*-p62 interplay may eventually contribute to the diagnosis and treatment of malignancies.

## MATERIALS AND METHODS

### Cell culture, reagents, and phenotypic assays

The FaDu, OC3, OECM1, SAS and SCC25 HNSCC cell lines, 293FT cells, phoenix package cell and HIOK established in our laboratory, were cultured as previously described [1, 4, 50]. For hypoxia culture, cells were grown in an atmosphere with 1% O_2_, 5% CO_2_, and 94% N_2_ for various time periods. The *miR-372* mimic, mirVana^TM^ *miR-372* inhibitor and controls were purchased from Applied Biosystems (Foster City, CA). They were optimized at 60 nM or 120 nM when used for cell treatment. The si-HIF1α, si-p62, si-NQO1 oligonucleotides, together with the scramble (si-Scr) control oligonucleotide, were purchased from Santa Cruz Biotech (Santa Cruz, CA) (Table S1). The cisplatin, ROS inhibitor NAC, CoCl_2_ and DMOG, which stabilize HIF1α, and the autophagy inhibitor 3MA were purchased from Sigma-Aldrich (St Louise, MO). Analysis of cell growth, migration, invasion, AIG and subcutaneous xenografic tumorigenesis of SAS cells followed various protocols that have been previously published [1, 3]. Unless specified, all other material was purchased from Sigma-Aldrich.

### Tissue and blood samples

The surgical specimens consisted of the primary HNSCC tumors together with paired NCMTs (Table S2). Macrodissection was performed to retrieve epithelial component from NCMT tissues. All HNSCC tissues were examined using frozen section to ascertain that they contained tumor cell component > 70%. Plasma and saliva (3 - 5 ml) were collected one week before surgery from patients, and around one month after surgery from patients who were available [9]. Blood samples were also collected from 18 sex- and age-matched control subjects without any head and neck disease. The above samples were collected after obtaining written informed consent and this study was approved by The Institutional Review Board in Mackay Memorial Hospital with approval number 11MMHIS026.

### Reporter construct and activity assays

A region consisting of the 3′UTR of the *p62* gene, predicted by TargetScan and PicTar software, which encompassed the *miR-372* binding site, was amplified by PCR. The amplicons were cloned into the pMIR-REPORT reporter vector (Applied Biosystems) to produce a WtR plasmid. A MutR plasmid was obtained from the WtR plasmid by replacing the sequence AGCACUU at the target site of *miR-372* with GAGCTCG in order to create a new *Sac*І restriction enzyme digestion site. Firefly luciferase activity normalized against renilla luciferase activity, which was used to represent transfection efficiency, is presented as reporter activity in this study.

### Establishment of cell subclones

The *pre-miR-372* sequence was cloned into a lentivirus vector carrying a red fluorescence (RFP) tag; this vector was purchased from Biosetta (San Diego, CA). The presence of red fluorescence in the cells indicated infection. Cell subclones with stable *miR-372* expression and controls were established by sorting of red fluorescence.

miRZip™ lentivector-based anti-*miR-372* plasmid, designated miRZip-372 in this study, was purchased from System Biosciences (Mountain View, CA). miRZip-Scr containing scramble sequence as the insert was a gift from Professor Yang, M-H. The presence of green fluorescence in the cells indicated infection. Cell subclones with stable *miR-372* inhibition and controls were established by puromycin selection.

The coding sequence (CDS) of p62 and the full-length p62 sequence including the CDS and 3′UTR, were cloned into the pBabe-puro vector to produce constructs. Cell subclones with stable p62 expression were established by viral infection and puromycin selection and were designated p62 CDS and p62 CDS+3′UTR, respectively [1].

Short hairpin sh-p62 constructs (Table S3) packed in lentiviruses were obtained from the RNA interference consortium (Academia Sinica, Taipei, Taiwan). Cell subclones exhibiting the knockdown of p62 (sh-p62) and sh-Luc control were achieved after viral infection and puromycin selection [1].

### Quantitative (q)RT-PCR

Total RNA from cell, tissue or plasma was reversed transcribed for qRT-PCR analysis. The expression of a panel of miRNAs, and the mRNA expression of *p62* and *NQO*1, were assessed using TaqMan kits (Applied Biosystems) according to procedures described previously [1, 9]. *RNU6B*, *miR-16* and *GAPDH* were used as the internal controls. The threshold cycle (Ct) method was used to measure the relative changes in expression. The −ΔΔCt was the difference of ΔCt values between different experimental settings and the sample groups. The 2^−ΔΔCt^ indicates fold of change in expression.

### Western blot analysis

Cell lysate (60 μg) was subjected to Western blot analysis using various primary antibodies (Table S4) and secondary antibodies (Table S5) according to previously described protocols [1].

### Immunostaining

For immunofluorescence (IF), cells or tissue tissues were incubated with primary antibodies (Table S4) and secondary antibodies (Table S5). The nuclei were stained with Hoechst 33258. Immunofluorescence was photographed using a confocal laser scanning microscope (Leica, Heidelberg, Germany). For immunohistochemistry (IHC), the tissue sections were incubated at 4°C for 16 h with the primary antibodies (Table S4) and then processed using a LSAB™2 kit (Dako, Glostrup, Denmark). Pre-immunized mouse IgG was used as a negative control.

### ROS detection

Measurement of ROS was carried out using 7′-dichlorodihydrofluorescein diacetate (H_2_DCFDA) fluorescence. An assay kit was used and the procedure followed the conditions recommended by the manufacturer (Molecular Probes, Eugene, OR). Values were obtained by subtracting background fluorescence as measured in the unstained controls without any loading of H_2_DCFDA. Fold changes of test group were achieved by normalizing to control group.

### Statistics

Mann-Whiney test, Wilcoxon signed rank test, two-way ANOVA test and correlation analysis were used to compare the differences among variants. The extent that the obtained –ΔCt could be used to distinguish disease status was determined using receiver operating characteristic (ROC) analysis; the area under the curve (AUC) was used to test discriminative ability. A *p* value of less than 0.05 was considered to be statistically significant.

Additional tables are presented in the supplementary material.

## SUPPLEMENTARY MATERIAL TABLES AND FIGURE



## References

[1] Liu CJ, Tsai MM, Hung PS, Kao SY, Liu TY, Wu KJ, Chiou SH, Lin SC, Chang KW (2010). miR-31 ablates expression of the HIF regulatory factor FIH to activate the HIF pathway in head and neck carcinoma. Cancer Res.

[2] Leemans CR, Braakhuis BJ, Brakenhoff RH (2011). The molecular biology of head and neck cancer. Nat Rev Cancer.

[3] Liu CJ, Shen WG, Peng SY, Cheng HW, Kao SY, Lin SC, Chang KW (2014). miR-134 induces oncogenicity and metastasis in head and neck carcinoma through targeting WWOX gene. Int J Cancer.

[4] Lu HH, Kao SY, Liu TY, Liu ST, Huang WP, Chang KW, Lin SC (2010). Areca nut extract induced oxidative stress and upregulated hypoxia inducing factor leading to autophagy in oral cancer cells. Autophagy.

[5] Al Moustafa AE, Foulkes WD, Benlimame N, Wong A, Yen L, Bergeron J, Batist G, Alpert L, Alaoui-Jamali MA (2004). E6/E7 proteins of HPV type 16 and ErbB-2 cooperate to induce neoplastic transformation of primary normal oral epithelial cells. Oncogene.

[6] Tu HF, Lin SC, Chang KW (2013). MicroRNA aberrances in head and neck cancer: pathogenetic and clinical significance. Curr Opin Otolaryngol Head Neck Surg.

[7] Bartel DP (2009). MicroRNAs: target recognition and regulatory functions. Cell.

[8] Esquela-Kerscher A, Slack FJ (2006). Oncomirs - microRNAs with a role in cancer. Nat Rev Cancer.

[9] Liu CJ, Kao SY, Tu HF, Tsai MM, Chang KW, Lin SC (2010). Increase of microRNA miR-31 level in plasma could be a potential marker of oral cancer. Oral diseases.

[10] Xing L, Todd NW, Yu L, Fang H, Jiang F (2010). Early detection of squamous cell lung cancer in sputum by a panel of microRNA markers. Mod Pathol.

[11] Roa WH, Kim JO, Razzak R, Du H, Guo L, Singh R, Gazala S, Ghosh S, Wong E, Joy AA, Xing JZ, Bedard EL (2012). Sputum microRNA profiling: a novel approach for the early detection of non-small cell lung cancer. Clin Invest Med.

[12] Kinoshita T, Hanazawa T, Nohata N, Kikkawa N, Enokida H, Yoshino H, Yamasaki T, Hidaka H, Nakagawa M, Okamoto Y, Seki N (2012). Tumor suppressive microRNA-218 inhibits cancer cell migration and invasion through targeting laminin-332 in head and neck squamous cell carcinoma. Oncotarget.

[13] Wu X, Bhayani MK, Dodge CT, Nicoloso MS, Chen Y, Yan X, Adachi M, Thomas L, Galer CE, Jiffar T, Pickering CR, Kupferman ME, Myers JN, Calin GA, Lai SY (2013). Coordinated targeting of the EGFR signaling axis by microRNA-27a*. Oncotarget.

[14] Vigneswaran N, Wu J, Song A, Annapragada A, Zacharias W (2011). Hypoxia-induced autophagic response is associated with aggressive phenotype and elevated incidence of metastasis in orthotopic immunocompetent murine models of head and neck squamous cell carcinomas (HNSCC). Exp Mol Pathol.

[15] Yang MH, Wu MZ, Chiou SH, Chen PM, Chang SY, Liu CJ, Teng SC, Wu KJ (2008). Direct regulation of TWIST by HIF-1alpha promotes metastasis. Nat Cell Biol.

[16] Semenza GL (2010). HIF-1: upstream and downstream of cancer metabolism. Curr Opin Genet Dev.

[17] Lin PY, Yu CH, Wang JT, Chen HH, Cheng SJ, Kuo MY, Chiang CP (2008). Expression of hypoxia-inducible factor-1 alpha is significantly associated with the progression and prognosis of oral squamous cell carcinomas in Taiwan. J Oral Pathol Med.

[18] Pouyssegur J, Dayan F, Mazure NM (2006). Hypoxia signalling in cancer and approaches to enforce tumour regression. Nature.

[19] Camps C, Buffa FM, Colella S, Moore J, Sotiriou C, Sheldon H, Harris AL, Gleadle JM, Ragoussis J (2008). hsa-miR-210 Is induced by hypoxia and is an independent prognostic factor in breast cancer. Clin Cancer Res.

[20] Du R, Sun W, Xia L, Zhao A, Yu Y, Zhao L, Wang H, Huang C, Sun S (2012). Hypoxia-induced down-regulation of microRNA-34a promotes EMT by targeting the Notch signaling pathway in tubular epithelial cells. PloS one.

[21] He M, Wang QY, Yin QQ, Tang J, Lu Y, Zhou CX, Duan CW, Hong DL, Tanaka T, Chen GQ, Zhao Q (2013). HIF-1alpha downregulates miR-17/20a directly targeting p21 and STAT3: a role in myeloid leukemic cell differentiation. Cell Death Differ.

[22] Loayza-Puch F, Yoshida Y, Matsuzaki T, Takahashi C, Kitayama H, Noda M (2010). Hypoxia and RAS-signaling pathways converge on, and cooperatively downregulate, the RECK tumor-suppressor protein through microRNAs. Oncogene.

[23] Voorhoeve PM, le Sage C, Schrier M, Gillis AJ, Stoop H, Nagel R, Liu YP, van Duijse J, Drost J, Griekspoor A, Zlotorynski E, Yabuta N, De Vita G, Nojima H, Looijenga LH, Agami R (2006). A genetic screen implicates miRNA-372 and miRNA-373 as oncogenes in testicular germ cell tumors. Cell.

[24] Yamashita S, Yamamoto H, Mimori K, Nishida N, Takahashi H, Haraguchi N, Tanaka F, Shibata K, Sekimoto M, Ishii H, Doki Y, Mori M (2012). MicroRNA-372 is associated with poor prognosis in colorectal cancer. Oncology.

[25] Cho WJ, Shin JM, Kim JS, Lee MR, Hong KS, Lee JH, Koo KH, Park JW, Kim KS (2009). miR-372 regulates cell cycle and apoptosis of ags human gastric cancer cell line through direct regulation of LATS2. Mol Cells.

[26] Li G, Zhang Z, Tu Y, Jin T, Liang H, Cui G, He S, Gao G (2013). Correlation of microRNA-372 upregulation with poor prognosis in human glioma. Diagn Pathol.

[27] Gu H, Guo X, Zou L, Zhu H, Zhang J (2013). Upregulation of microRNA-372 associates with tumor progression and prognosis in hepatocellular carcinoma. Mol Cell Biochem.

[28] Lee KH, Goan YG, Hsiao M, Lee CH, Jian SH, Lin JT, Chen YL, Lu PJ (2009). MicroRNA-373 (miR-373) post-transcriptionally regulates large tumor suppressor, homolog 2 (LATS2) and stimulates proliferation in human esophageal cancer. Exp Cell Res.

[29] Wong TS, Liu XB, Wong BY, Ng RW, Yuen AP, Wei WI (2008). Mature miR-184 as potential oncogenic microRNA of squamous cell carcinoma of tongue. Clin Cancer Res.

[30] Zhou AD, Diao LT, Xu H, Xiao ZD, Li JH, Zhou H, Qu LH (2012). beta-Catenin/LEF1 transactivates the microRNA-371-373 cluster that modulates the Wnt/beta-catenin-signaling pathway. Oncogene.

[31] Tian RQ, Wang XH, Hou LJ, Jia WH, Yang Q, Li YX, Liu M, Li X, Tang H (2011). MicroRNA-372 is down-regulated and targets cyclin-dependent kinase 2 (CDK2) and cyclin A1 in human cervical cancer, which may contribute to tumorigenesis. J Biol Chem.

[32] Moscat J, Diaz-Meco MT (2012). p62: a versatile multitasker takes on cancer. Trends Biochem Sci.

[33] Rosenfeldt MT, O'Prey J, Morton JP, Nixon C, MacKay G, Mrowinska A, Au A, Rai TS, Zheng L, Ridgway R, Adams PD, Anderson KI, Gottlieb E, Sansom OJ, Ryan KM (2013). p53 status determines the role of autophagy in pancreatic tumour development. Nature.

[34] Park MT, Oh ET, Song MJ, Lee H, Choi EK, Park HJ (2013). NQO1 prevents radiation-induced aneuploidy by interacting with Aurora-A. Carcinogenesis.

[35] Komatsu M, Kurokawa H, Waguri S, Taguchi K, Kobayashi A, Ichimura Y, Sou YS, Ueno I, Sakamoto A, Tong KI, Kim M, Nishito Y, Iemura S, Natsume T, Ueno T, Kominami E (2010). The selective autophagy substrate p62 activates the stress responsive transcription factor Nrf2 through inactivation of Keap1. Nat Cell Biol.

[36] Valencia T, Kim JY, Abu-Baker S, Moscat-Pardos J, Ahn CS, Reina-Campos M, Duran A, Castilla EA, Metallo CM, Diaz-Meco MT, Moscat J (2014). Metabolic reprogramming of stromal fibroblasts through p62-mTORC1 signaling promotes inflammation and tumorigenesis. Cancer Cell.

[37] Gandin V, Senft D, Topisirovic I, Ronai ZA (2013). RACK1 function in cell motility and protein synthesis. Genes Cancer.

[38] Garces JA, Clark IB, Meyer DI, Vallee RB (1999). Interaction of the p62 subunit of dynactin with Arp1 and the cortical actin cytoskeleton. Curr Biol.

[39] Huang S, Okamoto K, Yu C, Sinicrope FA (2013). p62/sequestosome-1 upregulation promotes ABT-263-induced caspase-8 aggregation/activation on the autophagosome. J Biol Chem.

[40] Pezeshkpour GH, Moatamed F, Lewis M, Hoang B, Rettig M, Mortazavi F (2013). CRK SH3N domain diminishes cell invasiveness of non-small cell lung cancer. Genes Cancer.

[41] Shibata A, Tanabe E, Inoue S, Kitayoshi M, Okimoto S, Hirane M, Araki M, Fukushima N, Tsujiuchi T (2013). Hydrogen peroxide stimulates cell motile activity through LPA receptor-3 in liver epithelial WB-F344 cells. Biochem Biophys Res Commun.

[42] Shi D, Guo W, Chen W, Fu L, Wang J, Tian Y, Xiao X, Kang T, Huang W, Deng W (2012). Nicotine promotes proliferation of human nasopharyngeal carcinoma cells by regulating alpha7AChR, ERK, HIF-1alpha and VEGF/PEDF signaling. PloS one.

[43] Jain A, Lamark T, Sjottem E, Larsen KB, Awuh JA, Overvatn A, McMahon M, Hayes JD, Johansen T (2010). p62/SQSTM1 is a target gene for transcription factor NRF2 and creates a positive feedback loop by inducing antioxidant response element-driven gene transcription. J Biol Chem.

[44] Yu H, Su J, Xu Y, Kang J, Li H, Zhang L, Yi H, Xiang X, Liu F, Sun L (2011). p62/SQSTM1 involved in cisplatin resistance in human ovarian cancer cells by clearing ubiquitinated proteins. Eur J Cancer.

[45] Galavotti S, Bartesaghi S, Faccenda D, Shaked-Rabi M, Sanzone S, McEvoy A, Dinsdale D, Condorelli F, Brandner S, Campanella M, Grose R, Jones C, Salomoni P (2013). The autophagy-associated factors DRAM1 and p62 regulate cell migration and invasion in glioblastoma stem cells. Oncogene.

[46] Puissant A, Fenouille N, Auberger P (2012). When autophagy meets cancer through p62/SQSTM1. Am J Cancer Res.

[47] Inui T, Chano T, Takikita-Suzuki M, Nishikawa M, Yamamoto G, Okabe H (2013). Association of p62/SQSTM1 excess and oral carcinogenesis. PloS one.

[48] Huang JS, Cho CY, Hong CC, Yan MD, Hsieh MC, Lay JD, Lai GM, Cheng AL, Chuang SE (2013). Oxidative stress enhances Axl-mediated cell migration through an Akt1/Rac1-dependent mechanism. Free Radic Biol Med.

[49] Lee SO, Chang YC, Whang K, Kim CH, Lee IS (2007). Role of NAD(P)H:quinone oxidoreductase 1 on tumor necrosis factor-alpha-induced migration of human vascular smooth muscle cells. Cardiovasc Res.

[50] Hung PS, Tu HF, Kao SY, Yang CC, Liu CJ, Huang TY, Chang KW, Lin SC (2014). miR-31 is upregulated in oral premalignant epithelium and contributes to the immortalization of normal oral keratinocytes. Carcinogenesis.

