# Supplementary material for: miR-372 inhibits p62 in head and neck squamous cell carcinoma in vitro and in vivo
Source: Oncotarget. 2015 Jan 21;6(8):6062–75. doi: 10.18632/oncotarget.3340 (PMC4467422; doi:10.18632/oncotarget.3340)
Supplement: Supplementary file 1 [file oncotarget-06-6062-s001.pdf]

miR-372 inhibits p62 in head and neck squamous cell carcinoma *in vitro* and *in vivo*

Supplementary Material

Supplementary Tables

Table S1: siRNA oligonucleotides used in the present study

siRNA	Supplier	Cat. No.
si-HIF1α	Santa Cruz Biotech	sc-35561
si-NQO1	Santa Cruz Biotech	sc-37139
si-p62	Santa Cruz Biotech	sc-29679
si-Scr	Santa Cruz Biotech	sc-37007

Table S2: Clinicopathological parameters of the HNSCC

n =	66
T1, 2	20
T3, 4	46
N0	40
N+	26
Stage I, II	17
Stage III, IV	49
Alive	41
Expired	15

Table S3: sh-RNA clones used in the present study

	Clone ID
sh-Luc	TRCN0000072249
sh-p62 (7234)	TRCN0000007234
sh-p62 (7235)	TRCN0000007235

Table S4: Primary antibodies used in the present study

Antibody	MW (kDa)	Host	Dilution	Supplier	Cat. No.
BNIP3	25	mouse	1:1000	Sigma-Aldrich	B7931
GAPDH	37	mouse	1: 10000	Santa Cruz Biotech	sc32233
HIF1α	120	mouse	1:1000	BD Biosciences	610959
NQO1#	31	rabbit	1:1000 (1: 200#)	Abcam	ab34173
p62*#	62	mouse	1:1000 (1: 100*#)	Santa Cruz Biotech	SC28359

*For IHC analysis
#For IF analysis

Table S5: Secondary antibodies used in the present study

Antibody	Dilution	Supplier	Cat. No.
anti-mouse	1:1000	Chemicon	AP124R
anti-rabbit	1:1000	Santa Cruz Biotech	Sc-2768
anti-mouse FITC*	1:200	Millipore	AP124F
anti-rabbit Texas Red*	1:200	Abcam	ab6719

*For IF analysis

Supplementary Figure

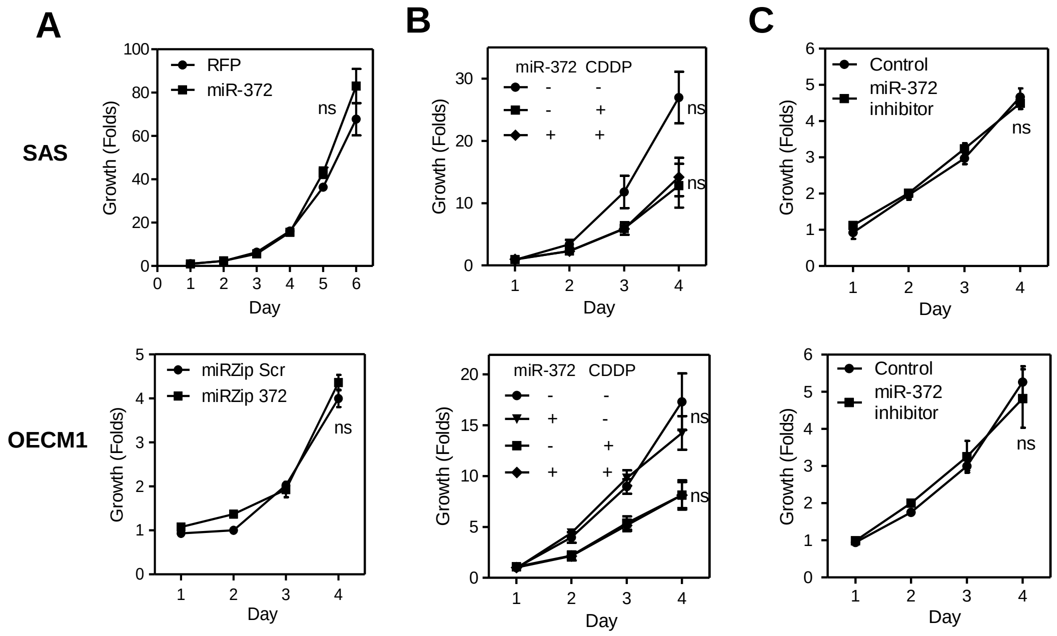

Fig. S1: Growth of HNSCC cells as related to *mR-372* expression. Upper, SAS cells. Lower, OECM1 cells. (A) SAS-miR-372 cell subclone exhibited no difference in growth relative to control cell subclone (SAS-RFP). OECM1-miRZip-372 cell subclone also exhibited no change of growth relative to control cell subclone (OECM1-miRZip-Scr). (B) IC_{50} dosages of Cisplatin (labeled as CDDP in illustration); predetermined to be 10 µM and 5 µM for SAS cells and OECM1 cells, respectively, in pilot studies. Exogenous *miR-372* had no influence on cell growth of SAS cells in the presence of 10 µM cisplatin. Exogenous *miR-372* expression had no influence on cell growth of OECM1 cells in the presence of or absence of 5 µM cisplatin (C) *miR-372* inhibition had no effect on the growth of HNSCC cells. Data shown were mean ± SE. *ns*, not significant. two-way ANOVA test.
